# miR-153-3p, a new bio-target, is involved in the pathogenesis of acute graft-versus-host disease via inhibition of indoleamine- 2,3-dioxygenase

**DOI:** 10.18632/oncotarget.10220

**Published:** 2016-06-22

**Authors:** Xiao-su Zhao, Yi-nuo Wang, Meng Lv, Yuan Kong, Hong-xue Luo, Xiao-yang Ye, Qi Wu, Tong-feng Zhao, Yue-huan Hu, Hong-yu Zhang, Ming-Rui Huo, Jun Wan, Xiao-jun Huang

**Affiliations:** ^1^ Peking University People's Hospital, Peking University Institute of Hematology, Beijing Key Laboratory of Hematopoietic Stem Cell Transplantation, Beijing, China; ^2^ Shenzhen Key Laboratory for Neuronal Structural Biology, Biomedical Research Institute, Shenzhen Peking University - The Hong Kong University of Science and Technology Medical Center, Shenzhen, China; ^3^ Peking-Tsinghua Center for Life Sciences, Beijing, China; ^4^ Department of Hematology, Peking University Shenzhen Hospital, Shenzhen, China; ^5^ Division of Life Science, The Hong Kong University of Science and Technology, Clear Water Bay, Hong Kong, China; ^6^ Collaborative Innovation Center of Hematology, Peking University, Beijing, China

**Keywords:** acute graft-versus-host disease, microRNA, allogeneic hematopoietic stem cell transplantation, indoleamine-2,3-dioxygenase, regulation

## Abstract

Acute graft-versus-host disease (aGVHD) is a major cause of morbidity and mortality after allogeneic hematopoietic stem cell transplantation. Therefore, seeking reliable biomarkers and delineating the potential biological mechanism are important for optimizing treatment strategies and improving their curative effect. In this study, using a microRNA polymerase chain reaction (PCR)-based chip assay, microRNA-153-3p (miR-153-3p) was screened and selected as a potential biomarker of aGVHD. The elevated plasma miR-153-3p levels at +7 d after transplant could be used to predict the upcoming aGVHD. The area under the receiver operating characteristic curve for aGVHD+/aGVHD- patients receiving haploidentical transplant was 0.808 (95% confidence interval, 0.686-0.930) in a training set and 0.809 (95% confidence interval, 0.694-0.923) in a validation set. Interestingly, bioinformatics analysis indicated that indoleamine-2,3-dioxygenase (IDO) is a potential target of miR-153-3p. *In vitro* study confirmed that IDO could be directly inhibited by miR-153-3p. In a GVHD model, recipient mice injected with a miR-153-3p antagomir exhibited higher IDO expression levels at the early stage after transplantation, as well as delayed aGVHD and longer survival, indicating that the miR-153-3p level at +7 d post-transplant is a good predictor of aGVHD. miR-153-3p participates in aGVHD development by inhibiting IDO expression and might be a novel bio-target for aGVHD intervention.

## INTRODUCTION

Acute graft-versus-host disease (aGVHD) is a major cause of morbidity and mortality after allogeneic hematopoietic stem cell transplantation (allo-HSCT). A timely diagnosis and treatments that are more targeted are essential for improving their therapeutic effects.

MicroRNAs (miRs) are small non-coding RNAs that inhibit gene expression via translational repression or induction of mRNA degradation [1, 2]. Recently, circulating miRs have been reported to be promising diagnostic biomarkers for various types of diseases such as cancer due to their high stability in the blood and in other bodily fluids [3–6]. In addition, a few studies have shown that miRs are involved in many immune responses. For example, CD4+ T cells undergo Th2 differentiation when miR-155 is not present [7, 8]. miR-146a is able to control innate immune cell and T cell responses [9]. Several studies have also demonstrated roles for miRs in the pathogenesis of aGVHD. miR-100 has been shown to inhibit GVHD-related neovascularization, which is a common pathomechanistic feature of GVHD and ischemia [10]. miR-155 and miR-146a have also been shown to be essential regulators of aGVHD in mice [11, 12]. However, these studies focused on elucidating the roles of miRs in a mouse model. Data regarding the function of these miRs in human GVHD are limited. Thus, we were interested in the potential involvement of other miRs in human GVHD pathogenesis. Using a miR polymerase chain reaction (PCR)-based chip assay, miR-153-3p was screened and selected as a potential biomarker of aGVHD. More interestingly, indoleamine-2,3-dioxygenase (IDO) was found to be a potential target protein of miR-153-3p through bioinformatics analysis.

IDO catalyzes the first and rate-limiting step in the catabolism of tryptophan, which is the essential amino acid for T cell proliferation. Many previous studies have demonstrated that IDO displays an immunosuppressive effect associated with tumor immunity, autoimmunity and chronic infection [13–15]. Jasperson et al. demonstrated that IDO expression greatly increased in the colon after allo-HSCT and that a lack of IDO led to increased colon GVHD injury and accelerated lethality [16]. In addition, our previous work has suggested that the plasma expression levels of IDO correlated with the severity of aGVHD and varied with the development of aGVHD [17]. Increased IDO levels may serve as a protective mechanism during aGVHD. However, how IDO is controlled remains unclear.

In the present study, we used *in vitro* and animal model experiments to first demonstrate that IDO could be directly inhibited by miR-153-3p and that this miR might participate in aGVHD by inhibiting IDO. Moreover, we showed that the plasma level of miR-153-3p at +7 days (d) after allo-HSCT served as a promising biomarker to predict the occurrence of aGVHD. Therefore, miR-153-3p is involved in the pathogenesis of aGVHD through inhibiting IDO and might represent a putative new bio-target for novel intervention strategies for aGVHD.

## RESULTS

### Screening for biomarkers of aGVHD

**Figure 1:**
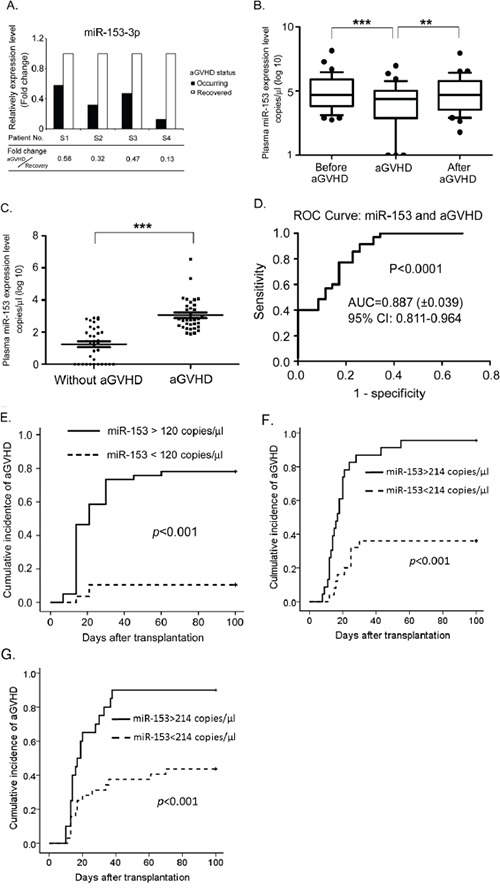
miR-153-3p is significantly increased when aGVHD occurs after allo-HSCT **A.** Four patients (numbers S1 to S4) who had aGVHD after allo-HSCT were selected for miR screening. Plasma samples were collected at two time points: during the occurrence of aGVHD and after recovery from aGVHD in response to treatment. Circulating RNA was purified, and qRT-PCR with SYBR Green was performed to detect the miR-153-3p expression level. The Y-axis shows the relative fold change in miR-153-3p during aGVHD compared with aGVHD recovery. **B.** In the aGVHD group, plasma samples were collected from all 30 patients at +7 d, +14 d, +21 d, +30 d, +45 d, +60 d, +90 d and the day of aGVHD occurrence. Absolute copies of miR-153-3p in the plasma were assessed using TaqMan qRT-PCR. miR-153-3p levels were compared with either those before aGVHD or those after aGVHD (aGVHD recovery) using the paired t-test. ***: p<0.0001; **: p<0.001. **C.** The expression level of miR-153-3p at +7 d in the aGVHD group was compared with that in the control group in the training set. ***: p<0.001. **D.** A receiver operating characteristic (ROC) plot was used to differentiate GVHD patients from controls in the training set. The data shown in C were used to draw the ROC plot. miR-153-3p yielded an AUC of 0.887 with a sensitivity of 74.3% and a specificity of 80.0% for forecasting aGVHD after allo-HSCT. **E.** The cumulative incidence of aGVHD between the high and low miR-153-3p expression groups in the training set (p<0.001). **F.** The ROC plot of 49 patients receiving haploidentical transplant was used to differentiate GVHD patients from controls in the training set with a sensitivity of 71.0% and a specificity of 94.0%. **G.** The ROC plot of 52 patients in the validation set was used to differentiate GVHD patients from controls with a sensitivity of 62.5% and a specificity of 85.0%.

To identify a panel of peripheral miR biomarkers of aGVHD after allo-HSCT, we selected four patients (patients S1 to S4) with severe aGVHD and collected plasma samples at two time points: the onset of aGVHD and the time point at which aGVHD was controlled. The circulating RNA in the plasma was isolated, and in total, forty-eight miRs that have been found to be present in human plasma were detected by quantitative real-time PCR (qRT-PCR). Among these miRs, miR-153-3p levels were reduced in the presence of aGVHD compared with the samples for which aGVHD was controlled after treatment. The fold change (onset of aGVHD/remission of aGVHD) ranged from 0.13 to 0.58 (Figure 1A).

### miR-153-3p expression varies with the development of aGVHD

To further confirm the change in miR-153-3p during aGVHD progression in human, we prospectively collected plasma samples from 70 consecutive patients (aGVHD+, n=35 vs aGVHD-, n=35) at different time points after allo-HSCT. The basic clinical characteristics of these 70 patients, who were used as a training set, are shown in Table 1. The detailed data about aGVHD in these 35 patients with aGVHD are shown in Supplemental Table S2. In the aGVHD group, 30 patients had decreased expression levels of miR-153-3p at the time of aGVHD occurrence compared to samples from the same patients prior to aGVHD onset (p<0.0001). In addition, 25 of the patients displayed a subsequent increase in miR-153-3p levels when aGVHD was controlled. Among the five patients with increased miR-153-3p levels during aGVHD, three of them also showed increased miR-153-3p levels after aGVHD was controlled (Figure 1B). The plasma miR-153-3p expression profiles of the six patients after allo-HSCT are shown in Supplemental Figure S1.

**Table 1:** Patient characteristics

	Training set n=70		P value for difference between aGVHD- and aGVHD+	Validation set n=52		P value for difference between aGVHD- and aGVHD+
Characteristics	aGVHD- n=35	aGVHD+ n=35		aGVHD- n=20	aGVHD+ n=32	
Sex, male/female, no.	21/14	18/17	0.470	6/14	12/20	0.580
Age at HSCT yr, median (range)	23 (5-52)	25 (5-54)	0.311	29(3-58)	28(6-59)	0.531
Diagnosis, no.			0.225			0.062
AML	19	13		5	19	
ALL	12	15		8	11	
CML	0	3		2	0	
MDS	3	2		4	1	
Others	1	2		1	1	
Disease status			0.172			0.228
Standard risk	28	32		15	29	
high risk	7	3		5	3	
Transplant type, no. (%)			<0.001			NA
Matched related	15	2		0	0	
Mismatched related	17	32		20	32	
Matched unrelated	3	1		0	0	
Conditioning, no. (%)			0.242			1.000
BU/CY	2	13		0	0	
TBI/CY	0	2		0	0	
BU/CY + ATG	32	18		18	27	
TBI/CY + ATG	1	2		2	3	
Source of graft, no. (%)			0.046			0.626
BM + PBSC	29	34		19	31	
PBSC	6	1		1	1	
Gender match			0.624			0.946
Male-male	15	11		10	18	
Female-female	8	7		3	5	
Male-female	6	10		5	7	
Female-male	6	7		2	2	
Blood type match			0.795			0.739
ABO compatibility	20	23		13	17	
Major ABO incompatibility	7	4		3	4	
Minor ABO incompatibility	5	5		2	6	
Major/minor incompatibility	3	3		2	5	
Infused CD3+ T cells (×10^6^/kg)	197.36 (34.28-357.24)	205.28 (29.67-414.56)	0.232	201.34 (25.42-478.34)	208.45 (32.13-341.48)	0.441
CD4/CD8 ratio in bone marrow	1.17(0.46-2.83)	1.35(0.56-3.41)	0.314	1.37(0.66-3.04)	1.28(0.50-3.90)	0.708
aGVHD, no. (%)						
Grade I–II	-	27 (77.1%)		-	24(75.0%)	
Grade III-IV	-	8 (22.9%)		-	8(25.0%)	

### High expression level of miR-153-3p at +7 d after allo-HSCT can predict the occurrence of aGVHD

At 7 d after allo-HSCT, none of the 70 patients had aGVHD. The expression level of miR-153-3p was much higher in the aGVHD group (range=77 to 3,389,977 copies/l plasma; Lg [copies/μl]=3.056±0.167, mean±SEM) compared to the group without aGVHD (range=0 to 817 copies/μl plasma; Lg [copies/μl]=1.253±0.181, p<0.0001). Notably, in the control group, the expression level of miR-153-3p was undetectable in 11 patients at +7 d, whereas miR-153-3p could be detected in the aGVHD group (Figure 1C). Furthermore, a receiver operating characteristic (ROC) analysis was used to evaluate the diagnostic accuracy of miR-153-3p at +7 d. The ROC curve showed that the AUC was 0.887 (95% confidence interval [95% CI], 0.811-0.964, p<0.0001, Figure 1D). According to the ROC analysis, the optimal cut-off value for miR-153-3p was 120 copies/μl. The 70 patients were divided into two groups based on this cut-off value. The cumulative incidence of aGVHD between these two groups was significantly different (p<0.001, Figure 1E). Univariate analysis revealed that a higher expression level of miR-153-3p at +7 d (>120 copies/μl, p<0.001), a younger age (patients less than 30 years old, p=0.031) and undergoing haploidentical transplantation (p=0.001) were associated with a higher incidence of aGVHD. In the subsequent multivariate analysis, only a higher expression level of miR-153-3p at +7 d and undergoing haploidentical transplantation were independent risk factors for aGVHD (Table 2).

**Table 2:** Results of the multivariate analysis for aGVHD

Characteristics	Hazard ratio	95% confidence interval	P value
High level of miR-153-3p	6.318	2.608-15.307	<0.001
Disease in standard risk	0.239	0.062-0.922	0.039
Haploidentical transplant	2.903	0.395-21.338	0.009

Among these patients, 49 of them received haploidentical transplant. We recalculated the cut-off value of miR-153 at +7 d in those 49 patients who were included in the training set and who underwent haploidentical transplant. Through ROC analysis, the optimal cut-off value was determined to be 214 copies/μl (AUC, 0.808; 95% CI, 0.686-0.930; p<0.001); the cumulative incidence of aGVHD between two groups of patients divided based on this value is shown in Figure 1F. To further confirm the effect of miR-153 expression at +7 d after transplantation on the occurrence of aGVHD, we employed another cohort of patients as the validation set (n=52, Table 1). All the patients in the validation set received haploidentical transplant. Then, we investigated whether this cutoff value was a good fit for patients in the validation set. Fifty-two patients were divided into two groups based on the threshold value of 214 copies/μl. As expected, there was a significant difference in the cumulative incidence of aGVHD between these two groups (AUC, 0.809; 95% CI, 0.694-0.923; p<0.001, Figure 1G).

### IDO expression is directly inhibited by miR-153-3p

**Figure 2:**
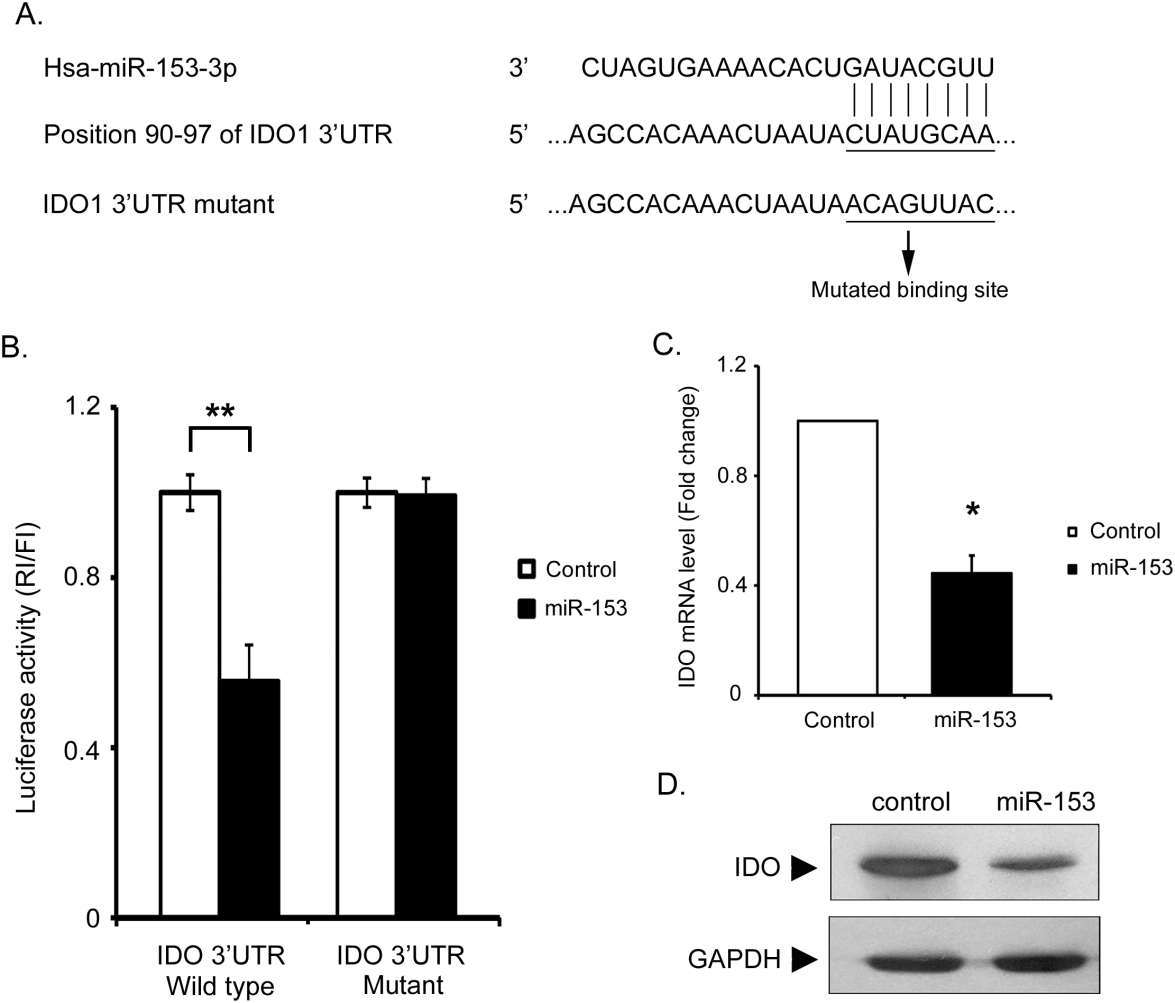
miR-153-3p can bind to IDO **A.** The unique site of complementarity in has-miR-153-3p and human IDO mRNA is CUAUGCAA. This binding site was mutated to ACAGUUAC for the luciferase activity assay. **B.** HEK-293T cells were transiently co-transfected with a combination of psiCHECK2 luciferase reporter plasmids encoding human IDO with a wild-type 3′UTR or mutated sequences and has-miR-153-3p mimic or mimic control. Luciferase activity was determined. The data represent the mean±SD (n=3). Normalization was performed with GAPDH. The blots shown are representative of 3 experiments. **: p<0.01. **C.** Total RNA isolated from HeLa cells transfected with has-miR-153-3p mimic or mimic control was subjected to real-time qPCR to assess the levels of IDO mRNA. The data represent the mean±SD (n=3). *: p<0.05. **D.** Twenty-four hours after HeLa cell transfection with has-miR-153-3p mimic or mimic control, the cells were induced with IFNγ for twelve hours and lysed in RIPA buffer. The lysates were subjected to Western blot analysis using IDO and GAPDH antibodies.

After miR-153-3p was identified as a potential biomarker of aGVHD, we were further interested in the role of this miR in GVHD progression. Bioinformatics analysis was conducted using TargetScan and miRanda tools. Human IDO, which has been shown to function in immunosuppression, was identified as a potential target of miR-153-3p. To investigate whether miR-153-3p could directly inhibit the expression of IDO, the wild-type 3′UTR of the IDO sequence and a mutant IDO 3′UTR containing a mutation in the miR-153-3p binding site were ligated into a luciferase dual-reporter plasmid (Figure 2A). The constructs were transfected into 293T cells together with miR-153-3p mimic or miR control mimic. The luciferase activity assay showed that miR-153-3p overexpression clearly decreased luciferase expression in the 293T cells compared with the controls (Figure 2B). However, luciferase activity was abolished in the cells containing the plasmid with the mutant 3′UTR IDO mRNA as the reporter construct (Figure 2B). Following miR-153-3p transfection in HeLa cells, both the IDO mRNA and protein levels were significantly reduced (Figure 2C and 2D), indicating that miR-153-3p could bind to the IDO 3′UTR and inhibit the expression of IDO.

### miR-153-3p and IDO expression correlates with aGVHD in murine recipients

**Figure 3:**
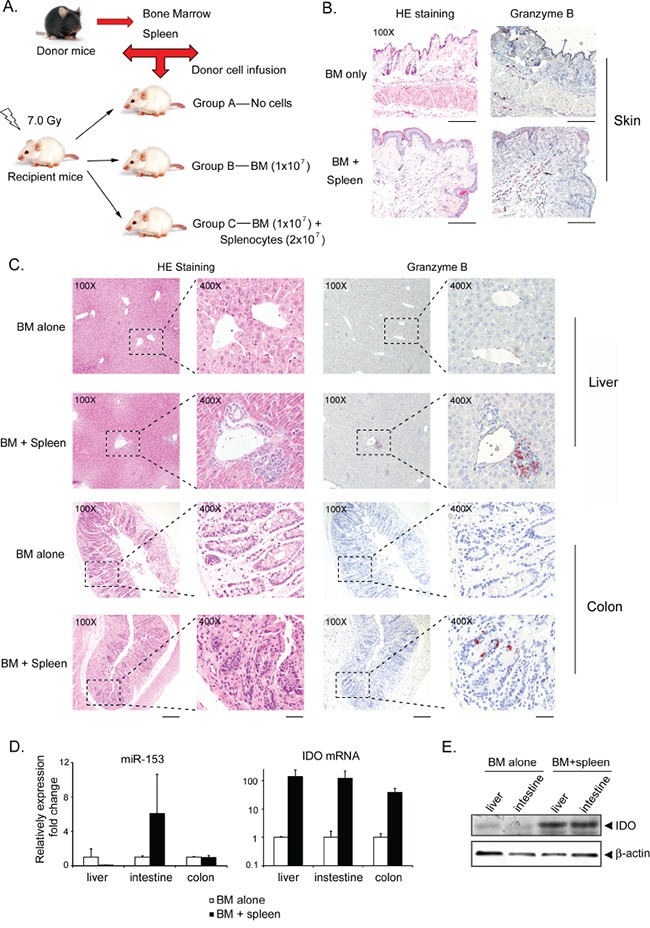
miR-153-3p expression is decreased while IDO expression is increased in murine recipients with aGVHD **A.** Schematic showing the aGVHD murine model used. **B-C.** Histopathologic evaluation of representative skin (B), liver and colon (C) samples collected from a mouse with a clinical GVHD score of more than or equal to 6. At least 3 mice in each group were collected for staining. The sections were stained either with hematoxylin and eosin (original magnification: 100×, left) or with anti-granzyme B antibody (original magnification: 100× or 400×, right). Red staining indicates granzyme B-positive cells. Error bar: 200 μm for 100× and 50 μm for 400×). **D.** Total RNA was isolated from liver, small intestine and colon tissues of at least three mice in each group. Mmu-miR-153 and IDO were quantified by TaqMan qRT-PCR. **E.** The IDO expression levels in the liver and small intestine were determined by immunoblotting; β-actin was used as the internal control.

To investigate whether miR-153-3p expression is decreased during aGVHD, a major histocompatibility complex (MHC)-mismatched HSCT model was used in which spleen cells (2×10^7^) and bone marrow cells (BMCs, 1×10^7^) from C57BL/6 (H-2^b^, B6) donors were transferred intravenously into lethally irradiated BALB/c (H-2^d^) (F1) recipient mice (Figure 3A). Two additional groups were included as controls: one group received no cell infusion (irradiation only), and the other group received only BM. The mice that received BM with or without spleen cells displayed successful engraftment at +7 d (Supplemental Figure S2A). The mice that received donor BM cells plus spleen cells (n=10) developed severe aGVHD that was confirmed by either clinical signs (Supplemental Figure S2B) or skin, liver and colon histology based on positive staining with the cytotoxic T cell marker granzyme B (Figure 3B-3C). Mice were killed at +7 d, +14 d and +21 d after infusion. The mice that received BM plus spleen cells achieved a clinical GVHD score equal to or more than 7 (median time, 12 d post-transplant; range, 7-15 d). Total RNA was extracted from the liver, colon, small intestine and spleen. At +21 d, miR-153-3p levels had decreased in the liver but significantly increased in the small intestine. Furthermore, IDO mRNA and protein levels were increased in both the liver and small intestine (Figure 3D, 3E).

### Recipient mice injected with a miR-153-3p antagomir exhibit delayed aGVHD and longer survival

**Figure 4:**
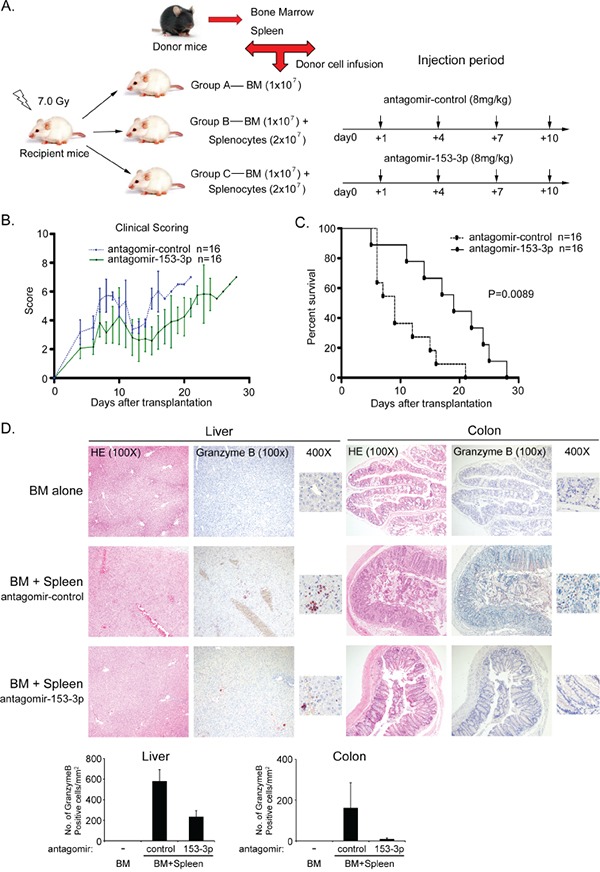
Recipient mice of an antagomir of miR-153-3p do not develop severe aGVHD and have increased survival **A.** Schematic of the different antagomir infusions used in the murine model of aGVHD. **B.** Clinical scores for the different recipient mouse groups after transplantation. **C.** Survival rate of aGVHD mice that received different antagomirs. **D.** Histopathologic evaluation of the liver and colon of different aGVHD mice at +21 d after allo-HSCT. HE and granzyme B staining were as the same as that in Figure 3. Granzyme B-positive cell numbers per mm^2^ were counted in at least 5 random fields.

To confirm the relationship between miR-153-3p and aGVHD, we performed the MHC-mismatched murine experiment as previously described. Three groups were included in this experiment: one group received BM only, and two groups received BM plus spleen cells via intravenous infusion in the tail of antagomir-control or antagomir-153-3p (8 mg/kg) at +1 d, +4 d, +7 d and +10 d (Figure 4A). Both groups that received BM plus spleen cells developed aGVHD. The incidence and severity of aGVHD significantly decreased in recipients injected with antagomir-153-3p infusion, as evidenced by their clinical GVHD scores (p< 0.0001, Figure 4B). Recipients with antagomir-153-3p infusion survived longer compared to those with antagomir-control infusion after transplantation (log-rank test, p=0.0089, Figure 4C). The pathological severity of aGVHD at +21 d in the liver and colon was also reduced in recipients who received antagomir-153-3p with positively stained granzyme B cell proportions as a marker (Figure 4D).

### Recipient mice of an antagomir of miR-153-3p display relatively higher IDO expression at the early stage after transplantation

**Figure 5:**
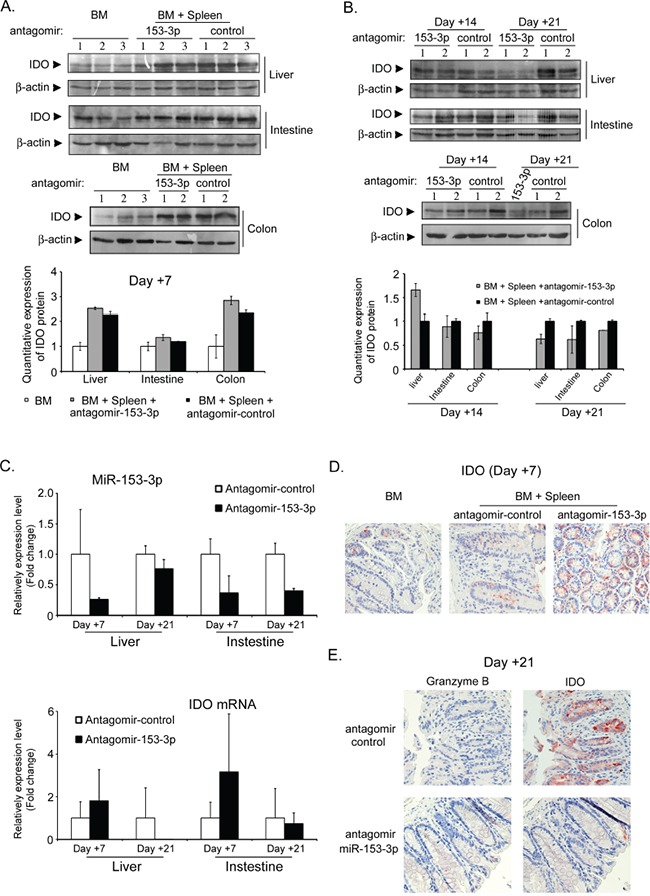
The miR-153-3p antagomir increases IDO expression at the early stage after allo-HSCT **A.** IDO expression levels in the liver, small intestine and colon were detected by immunoblotting at +7 d after allo-HSCT. The lower histogram shows the quantitative analysis. **B.** IDO expression levels in different tissues at +14 d and +21 d after allo-HSCT. **C.** Mmu-miR-153-3p and IDO mRNA levels were evaluated in the liver and small intestine by qRT-PCR at +7 d and +21 d after allo-HSCT. **D-E.** Granzyme B and IDO immunohistochemistry of colon tissues at +7 d (D) and +21 d (E) after allo-HSCT.

To further confirm whether the antagomir-153-3p increased IDO expression in different mouse tissues, we collected liver, colon, small intestine and spleen tissues at +7 d, +14 d and +21 d after transplantation. At +7 d, IDO was higher in different tissues of the antagomir-153-3p recipients (Figure 5A). At +14 d, the IDO expression level in antagomir-153-3p recipient spleens is also much higher than that in antagomir-control spleens, indicating that a high level of IDO in the spleen protect against aGVHD development (Supplemental Figure S2C). However, IDO expression increased in antagomir-control recipients over time until it was higher than that detected in antagomir-153-3p recipients, although the expression of miR-153-3p was continuously inhibited by antagomir (Figure 5B-5C). The immunohistochemical staining showed that at +7 d, the antagomir-153-3p recipients had a very high expression level of IDO in colon epithelial cells (Figure 5D). But, at +21 d, the IDO expression level in the colon cells of antagomir-153-3p recipients was significantly lower than that in the control group (Figure 5E), which indicated that the high expression level of IDO had a protective effect on aGVHD progression.

### IDO expression is lower in the aGVHD group at +7 d after allo-HSCT in humans

**Figure 6:**
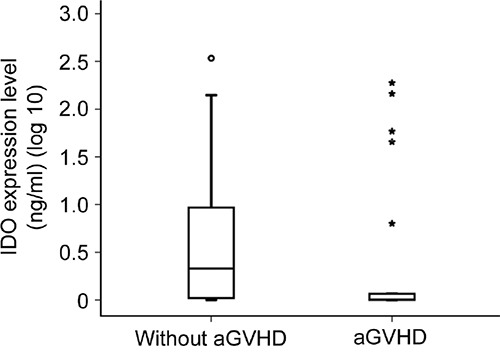
Expression level of IDO at +7 d in the aGVHD group and the control group (p=0.007)

Because miR-153-3p directly binds IDO, we further investigated whether the expression level of miR-153-3p correlated negatively with the expression level of IDO in humans. The plasma IDO level at +7 d in twenty-one patients in either the aGVHD group or the control group was examined by ELISA. The median expression level of IDO in the aGVHD group was 0.007 ng/ml (range, 0-2.276 ng/ml), while it was 0.339 ng/ml (range, 0-2.533 ng/ml) in the control group (p=0.007, Figure 6). As anticipated, the expression level of miR-153-3p in the aGVHD group (270 copies/μl [range, 0-5221 copies/μl] vs 28 copies/μl [range, 0-838 copies/μl], p=0.031) was much higher than that in the control group.

## DISCUSSION

miR-153 has mainly been reported to be a critical regulator in the neural molecular network [18, 19], and it is also related to tumor proliferation [20, 21]. In the present study, IDO, which is able to suppress T-cell immunity, was identified as a novel target of miR-153. It indicated that miR-153 might be related to some immune-related diseases, including GVHD. The majority of the miRs related to aGVHD have been shown to function in T or B lymphocytes [11, 12, 22]. However, IDO might inhibit aGVHD development through various types of cells in addition to lymphocytes, such as dendritic cells (DCs), mesenchymal stem cells (MSCs) and myeloid-derived suppressor cells (MDSCs). Waller et al. demonstrated that IDO expression by donor pDCs could inhibit continued T cell activation and GVHD [23]. It has also been reported that MSC infusion for the treatment of steroid-resistant GVHD is controlled by the induction of IDO [24]. In addition, one of the human MDSC subsets, CD14+HLA-DR^low/neg^ cells, was able to suppress the proliferation of autologous T cells in an IDO-dependent manner in patients with aGVHD [25]. Thus, the direct inhibition of IDO by miR-153-3p would provide a new mechanism by which miR-153 participates in the pathogenesis of aGVHD in cells other than lymphocytes.

Circulating miRs have been increasingly used as a new type of biomarker for various types of diseases because they are stable in peripheral blood and can be easily examined using noninvasive measures [2–4, 26, 27]. A study published in Blood has shown that a model that includes 4 miRs (miR-423, miR-199a-3p, miR-93, and miR-377) could predict the probability of aGVHD with an AUC of 0.80 [28]. However, they did not indicate the potential target genes of these selected miRs. In the current study, we first demonstrated that the plasma miR-153-3p level would be a good biomarker for predicting subsequent aGVHD. In addition, we revealed the direct target protein of miR-153-3p, IDO, which will be important for providing novel ideas for aGVHD therapy.

In this study, we found that the levels of miR-153-3p at +7 d in the GVHD group were higher than were those in the non-GVHD group. Accordingly, the expression level of IDO in the GVHD group was lower than that in the non-GVHD group. We speculated that this low level of IDO expression prevents the maintenance of immunotolerance after transplantation, thus leading to aGVHD. Consistent with this conclusion, Jasperson et al. demonstrated that the purposeful induction of IDO pre-transplantation by a TLR7/8 agonist could reduce subsequent GVHD lethality in a mouse model. An early increase in IDO expression may be particularly effective for reducing the overall T cell burden during the most intense inflammatory phase of conditioning [16]. Hence, the maintenance of immune tolerance during early stages after transplantation is very important for the prevention of aGVHD. Similarly, the work of Stickel et al. indicated that the expression of miR-146a, which is able to suppress T cell responses, decreased at early stages after allo-HSCT in mice that developed GVHD [12]. In addition, the changing trend of the corresponding IDO level was consistent with our previous results [17]. At the initiation of aGVHD, IDO increases potentially due to a protective negative feedback response of the body. A French study showed that the proportion of CD4+IDO+ T cells was significantly higher in patients with moderate gastrointestinal GVHD [29]. However, the increase in IDO finally determined the severity of aGVHD. The development of severe aGVHD might be due to an insufficient increase in IDO or exhaustion of the negative feedback loop.

The results of the GVHD mouse model experiments might elucidate the mechanism underlying the reduced IDO levels in early post-transplantation patients in response to endogenous miR-153 as a risk factor for aGVHD. However, this difference in endogenous miR-153 remains unclear. Recently, a single nucleotide polymorphism (SNP) of miR-146a has been demonstrated to reduce its expression level [12, 30]. Therefore, we assumed that a SNP might be responsible for the differences in endogenous miR-153 expression. Because receiving a haploidentical transplant is also an independent risk factor of aGVHD, there is also a possibility that the interaction between recipient and donor components might lead to the differential expression of miR-153 at the early stage after transplant. Mice that received the antagomir-control showed a gradual increase in IDO during aGVHD development that was even higher than the levels in mice that received the antagomir-153-3p. This result indicated that aGVHD induced IDO expression via a negative feedback mechanism. However, the miR-153-3p level in control mice remained relatively low in comparison to the antagomir group. This finding suggested that molecules other than miR-153 might control IDO during the late stages of transplantation. Based on the above results in animals, we speculated that the occurrence of aGVHD was determined by the early expression level of endogenous miR-153, although miR-153-3p might be only one of the inhibitors of IDO during aGVHD progression. Other regulatory mechanisms underlying the change in IDO levels likely exist, especially for different degrees and types of aGVHD.

In the present study, we were also interested in determining how circulating miR-153-3p inhibits intracellular IDO expression. Previous studies have demonstrated that miR can exist in a miR-induced silencing complex (miRISC). miRISC is able to fuse with vesicles and the endoplasmic reticulum or shuttle between intracellular and extracellular compartments. The miRs that are exported into the circulation are present in some types of exosomes. Zhang et al. reported that THP-1 cells could actively secrete miR-150. Subsequently, the secreted miR-150 was taken up by co-cultured microvascular endothelial cells, in which miR-150 inhibited the expression of c-MYB [31]. Thus, we presumed that circulating miR-153-3p might also function in a manner similar to those mentioned above in DCs, MSCs and MDSCs during GVHD. However, whether a specific type of cell actively secretes miR-153-3p and what controls its shuttling are still unknown. Future studies will delineate the exact regulatory mechanism underlying the effect of the circulating miR on intracellular proteina using *in vitro* or *in vivo* experiments.

In conclusion, this study is the first to show that IDO is a new target of miR-153-3p. Animal model experiments suggested that miR-153-3p might participate in aGVHD by inhibiting IDO expression. Clinically, elevated levels of plasma miR-153-3p at +7 d after transplantation would be a good predictor of the onset of aGVHD. Therefore, miR-153-3p might be a putative bio-target for novel intervention strategies for aGVHD. The elucidation of other mechanisms that inhibit IDO during aGVHD requires further study.

## MATERIALS AND METHODS

### Patient samples

Human plasma samples from consecutive patients who underwent allo-HSCT from Sep 2012 to Jun 2013 (training set), Sep 2014 to Nov 2014 (validation set) were collected from Peking University Institute of Hematology. All of the patients provided informed consent. This study was approved by the Institutional Review Board of Peking University People's Hospital. Plasma samples were prospectively collected from patients at +7 d, +14 d, +21 d, +30 d, +45 d, +60 d, and +90 d after allo-HSCT and at the occurrence of aGVHD. Finally, 70 patients (training set) and 52 patients (validation set) whose plasma samples were all available at the time points mentioned above were enrolled in this study. The characteristics of these two sets of patients are summarized in Table 1. Patients with acute leukemia in the third complete remission (CR3) or beyond, those in non-remission, and patients with chronic myeloid leukemia in the blast or accelerated phase were classified as advanced stage.

### Transplant protocols

All of the patients in the present study received myeloablative conditioning regimens. Transplantations were performed as previously described [32, 33]. Patients who received human leukocyte antigen (HLA) were matched to related transplant patients who received busulfan (BU, 0.8 mg/kg iv, q6h) and cyclophosphamide (CTX, 1.8 g/m^2^/d for 2 d) or total body irradiation (TBI, 7.7 Gy) administered as one fraction, followed by CTX. Patients who received HLA-mismatched transplants from a relative and HLA-matched transplants from unrelated donors were conditioned with BU+CTX+human antithymocyte globulin (ATG) or TBI+CTX+ATG (2.5 mg/kg/d iv for 4 d) (Lyon, France). All of the patients received G-CSF-mobilized BM and a peripheral blood stem cell transfusion followed by cyclosporine (CSA), mycophenolate mofetil (MMF) and short-term methotrexate (MTX).

### Mouse transplantation model

Eight- to twelve-week-old BALB/c (H-2^d^) and C57BL/6 (H-2^b^) mice were purchased from Vital River Laboratories. The mice were bred and maintained at the animal care facility of Peking University People's Hospital. Recipients (BALB/c mice) were supplied with unsterilized food and water *ad libitum*. Gentamicin (32×10^4^ U/L) and erythromycin (250 ng/L) were added to the water 7 d prior to HSCT to facilitate gastrointestinal preparation. The Institutional Review Board of Peking University People's Hospital approved this study (No. 2013-42).

All of the recipient BALB/c (H-2^d^) were irradiated (7.0 Gy total body irradiation), rested for 3 hours, and injected with donor BMCs (1×10^7^) and spleen cells (SCs, 2×10^7^) via the tail vein on day 0. Recipients were randomly assigned to one of three groups: group A (n=10), injected with BMCs only; group B (n=16); and group C (n=16).

Engraftment was assessed by examining the percentage of H-2Db (donor) cells in the BM of the recipients by fluorescence-activated cell sorting (FACS) analysis using an LSRFortessa (BD) at +7 d and +14 d after transplantation. The cell compositions of the donor BM and spleen grafts were examined by labeling with the following antibodies: CD3-V500, CD4-FITC, and CD8-APC (eBioscience, USA). Briefly, the cells were incubated with the antibody combinations for 20 minutes at 4°C. Erythrocytes were lysed in lysing solution twice for 10 minutes each. After the cells were washed with PBS, they were resuspended and analyzed by FACS. T cell subgroups were identified using antibodies and expressed as a percent of the positive cells within the nucleated cell population.

### Clinical and histological assessment of GVHD

Recipient mice were weighed 4 times a week and monitored twice daily for clinical signs of aGVHD and survival. GVHD scoring was performed according to Cooke et al. and included five clinical parameters: weight loss, posture (hunching), activity, fur texture, and skin integrity. On +7 d, three mice were randomly killed in each group to examine the plasma miR-153 expression level. The remaining mice who reached an aGVHD score of more than 6 were very sick and were killed. Small intestine, colon, skin, liver and spleen samples were collected on +7 d, +14 d and +21 d, and slides were prepared. The samples were stained with hematoxylin/eosin and scored based on a published histopathology scoring system by experienced pathologists who were blinded to the treatment groups.

### *In vivo* miR-153 antagomir treatment

Antagomirs were synthesized by Guangzhou RiboBio Co., Ltd. The antagomir sequences were as follows: antagomir-153, 5′-GAUCACUUUUGUGACUAUGCAA-3′; antagomir control, 5′-CAGUACUUUUGUGUAGUACAAA-3′. The animals were administered 8 mg/kg antagomir via tail vein injection on +1 d, +4 d, +7 d, +10 d after allo-HSCT.

### Statistical analysis

The data were analyzed using the SPSS 19.0 software package (IBM Corporation, Armonk, NY, USA). The results for miR-153-3p at +7 d after HSCT were log-transformed due to their abnormal distribution. Differences in animal survival were analyzed by the log-rank test. The statistical significance of the plasma miR-153-3p levels at +7 d after HSCT between the two groups was analyzed using Student's *t* test. p<0.05 was considered statistically significant. ROC analysis was used to evaluate the diagnostic accuracy of miR-153-3p at +7 d. To confirm outcomes and to adjust for potential confounding factors, multivariate Cox proportional hazards models were assessed for the proportional hazards assumption and for testing interaction terms with covariates. The following variables were included in the models: age (continuous, less than vs. greater than or equal to the median), sex, donor-patient sex match (female to male vs. others), transplant type, ABO match (identical+minor vs. major+bidirectional), HLA-match (one locus vs. two locus vs. three locus), disease status (high-risk [HR] vs. standard risk [SR]), CD3 dose (continuous, less than vs. greater than or equal to the median), and CD4/CD8 ratio in BM (continuous, less than vs. greater than or equal to the median).

## SUPPLEMENTARY MATERIALS TABLES AND FIGURES


