# Supplementary material for: miR-153-3p, a new bio-target, is involved in the pathogenesis of acute graft-versus-host disease via inhibition of indoleamine- 2,3-dioxygenase
Source: Oncotarget. 2016 Jun 22;7(30):48321–34. doi: 10.18632/oncotarget.10220 (PMC5217020; doi:10.18632/oncotarget.10220)
Supplement: Supplementary file 1 [file oncotarget-07-48321-s001.pdf]

miR-153-3p, a new bio-target, is involved in the pathogenesis of acute graft-versus-host disease via inhibition of indoleamine-2,3-dioxygenase

SUPPLEMENTARY MATERIALS AND METHODS

RNA isolation

Plasma RNA was isolated from a fixed volume of 200 µl of plasma from the patients on +7 d, +14 d, +21 d, +30 d, +45 d, +60 d and +90 d after HSCT. RNA extraction, quality control and normalization control methods were based on a previous study [1, 2]. Briefly, plasma RNA was isolated using TRI Reagent (Sigma) following the manufacturer's protocol. To normalize for some of the technical variability in the plasma RNA extraction, 10 µL of a pool of synthetic *C. elegans* miRNAs, cel-miR-54 and cel-miR-238 (synthetic RNA oligonucleotides synthesized by RIBOBIO, China) were added (0.1 ng of each oligonucleotide) to each sample after the initial plasma denaturation. The RNA was dissolved in 30 µL of RNase-free water.

Total RNA from cells or mouse tissues was extracted using TRI Reagent (Sigma). The RNA concentrations were determined using a NanoDrop spectrophotometer (Thermo Scientific).

Real-time quantitative PCR

Plasma miRNA levels were quantified using TaqMan miRNA qRT-PCR assays as previously described [2]. A fixed volume of 5 µL RNA was reverse transcribed using the Goscript Reverse Transcription System (Promega) and miRNA-specific stem-loop primers (Invitrogen) in a 20-µL RT reaction system. Real-time PCR reactions were performed using a CFX96TM Real-Time PCR detection system (Bio-Rad). Briefly, the reactions consisted of 0.7 µL cDNA and 0.2 µM of Taqman probe. All of the reactions were performed in triplicate. Absolute copies of miR-153-3p were calculated and normalized based on the standard curve. The standard curves were generated by ten-fold serial dilutions of synthetic has-miR-153 (synthesized by RIBOBIO, China) from 10^2 to 10^{13} copies. The levels of the synthetic miRNAs were assessed by RT-qPCR assay. The resulting Ct values were plotted versus the log of the amount of synthetic miRNAs. Absolute copies of miR-153-3p from plasma were calculated based on the standard curve and then multiplied by a normalization factor generated from the spiked *C. elegans* control and a selected endogenous control (has-miR-486) that was selected for its high levels and constant expression in plasma. The normalization factor was calculated for each sample based on the following formula: normalization factor=1/[2^(Median_Control_Ct value)−(Control_Average_Ct value of the given sample).

IDO mRNA expression was detected by SYBRGreen-based qRT-PCR. The relative expression was calculated using the comparative Ct method ($2^{-\Delta\Delta Ct}$). Each value was adjusted using GAPDH as the appropriate reference.

Cell culture and transfection

HeLa cells and HEK293T cells (ATCC) were cultured in DMEM (Invitrogen) supplemented with 10% FBS (GIBCO). Plasmids and miRNAs were transfected into HeLa or HEK293T cells using Lipofectamine 2000 reagent (Invitrogen) according to the manufacturer's instructions.

Constructs and luciferase reporter assay

The potential regulatory target of miR-153-3p was identified using bioinformatics analysis with TargetScan and Miranda. Human IDO 3'UTR was amplified from a cDNA library of human peripheral blood leukocytes using the following primers: forward, 5'-CTTTGTTTAAACTG TAACCCAACAAGAGCA-3'; reverse, 5'-GAATGCGG CCGCTCTTTAGCCCAGGCCGGATT-3'. The miR-153-3p binding site CTATGCAA was mutated to ACAGTTAC using the following primers: forward, 5'-CTAATAACA GTTACTGTTTTACCAATAATGC-3'; reverse, 5'-AA CAGTAACTGTTATTAGTTTGTGGCTCT-3'. The cDNA fragments were inserted into a luciferase dual-reporter plasmid psiCHECK™-2 Vector (Promega). HEK293T cells were seeded into 35-mm wells (1 x 10^5/well) and incubated overnight followed by transfection with various plasmids and miRNAs. Twenty-four hours after transfection, the cells were lysed, and the luciferase activity was measured using the Dual Luciferase Reporter Gene Assay Kit (Beyotime, China).

Preparation of cell extracts and western blot analysis

HeLa cells were transfected with 50 nM miR-153-3p mimic or miR mimic control for twelve hours followed by treatment with 1 ng/ml INF-gamma (Peprotech). Twenty-four

hours after the treatment, the cells were harvested and lysed in radioimmunoprecipitation assay (RIPA) lysis buffer [150 mM NaCl, 1% (v/v) Nonidet P-40, 0.1% (w/v) SDS, 0.5% deoxycholic acid, 1 x complete protease inhibitor cocktail (Roche)] in 50 mM phosphate buffer, pH 8.0. The proteins were separated by SDS-PAGE and then transferred onto a PVDF membrane (PALL) and probed with IDO antibody (Eptitomics) and GAPDH antibody (Cell Signaling). The proteins were visualized using the Supersignal West Pico Chemiluminescent Substrate kit (Pierce).

Immunohistochemistry

Mouse tissues were formalin-fixed followed by paraffin embedding. The embedded sections were examined by hematoxylin and eosin staining. Granzyme B and IDO immunostains were performed using rabbit polyclonal granzyme B antibody (Genetex) and polyclonal IDO antibody (Novus Biologicals), respectively, and visualized using an AEC kit.

Determination of plasma IDO

Plasma IDO levels were determined using a highly specific and sensitive human indoleamine 2,3-dioxygenase ELISA kit (WuHan Huamei Biotech Co., LTD) according to the manufacturer's instructions. Briefly, 100 µl of patient plasma was transferred into 96-well plates coated with IDO antibody and incubated for 2 h at 37°C. After the wells were washed, 100 µl of HRP-labeled monoclonal antibody was pipetted into the wells, and the plate was incubated for 1 h at 37°C. Next, 90 µl of substrate solution was added to each well, and the plate was incubated in the dark at room temperature for 20 minutes. After the addition of 50 µl of stop solution to each well followed by 10 seconds of agitation, the plate was read at 450 nm in a microplate reader. The sensitivity was 1 U/ml. All of the measurements were performed in duplicate wells.

REFERENCES

1. Kroh EM, Parkin RK, Mitchell PS, Tewari M: Analysis of circulating microRNA biomarkers in plasma and serum using quantitative reverse transcription-PCR (qRT-PCR). Methods 2010, 50:298-301.
2. Mitchell PS, Parkin RK, Kroh EM, Fritz BR, Wyman SK, Pogosova-Agadjanyan EL, Peterson A, Noteboom J, O'Briant KC, Allen A et al: Circulating microRNAs as stable blood-based markers for cancer detection. Proceedings of the National Academy of Sciences of the United States of America 2008, 105:10513-10518.

Supplementary Table S1: Sequences of primers and probes

Primer/probe name	Sequence
Hsa-miR-153 RT primer	5'-GTCGTATCCAGTGCGTGTCGTGGAGTCGGCAATTGCACTGGATAC GACGATCAC
Hsa-miR-153 Forward primer	5'-GCTTGCATAGTCACAAAAGTGATC
Hsa-miR-153 Taqman probe	5'-FAM-AGTCGGCAATTGCACTGGATACGAC-TAM
Hsa-miR-153 Reverse primer	5'-CGTATCCAGTGCGTGTCGT
Hsa-miR-486 RT primer	5'-GTCGTATCCAGTGCGTGTCGTGGAGTCGGCAATTGCACTGGATACG ACCTCGGG
Hsa-miR-486 Forward primer	5'-ACCGTCCTGTACTGAGCT
Hsa-miR-486 Taqman probe	5'-FAM-CACTGGATACGACCTCGGGGCAG-TAM
miR-universal Reverse primer	5'-GTGTCGTGGAGTCGGCAA
Cel-miR-54 RT primer	5'-GTCGTATCCAGTGCGTGTCGTGGAGTCGGCAATTGCACTGGATA CGACCTCGGA
Cel-miR-54 Forward primer	5'-GCAGACTCGTACCCGTAA
Cel-miR-54 Taqman probe	5'-FAM-CACTGGATACGACCTCGGATTATG-TAM
Cel-miR-238 RT primer	5'-GTCGTATCCAGTGCGTGTCGTGGAGTCGGCAATTGCACTGGATA CGACTCTGAA
Cel-miR-238 Forward primer	5'-AGCCTTTGTACTCCGATGC
Cel-miR-238 Taqman probe	5'-FAM-CACTGGATACGACTCTGAATGGCA-TAM
Human IDO Forward primer	5'- TGGCCAGCTTCGAGAAAGAG
Human IDO Reverse primer	5'- TGGCAAGACCTTACGGACATC
Mouse IDO Forward primer	5'- ATGTGGGCTTTGCTCTACCA
Mouse IDO Reverse primer	5'- CAGTGTGGGCAGCTTTTCAA
Mouse IDO Taqman probe	5'-FAM-CACTGGTGGAGCTGCCCGACGC-TAM
Human GAPDH Forward primer	5'-CAGCCTCAAGATCATCAGCA
Human GAPDH Reverse primer	5'-TGTGGTCATGAGTCCTTCCA
Mouse GAPDH Forward primer	5'-AACTTTGGCATTGTGGAAGG
Mouse GAPDH Reverse primer	5'-GGATGCAGGGATGATGTTCT
Mouse GAPDH Taqman probe	5'-FAM- CATTGTGGAAGGGCTCATGGTATGTAGG-TAM
U6 Forward primer	5'- GCTTCGGCAGCACATATACTAAAAT
U6 Reverse primer	5'- CGCTTCACGAATTTGCGTGTCAT
U6 Taqman probe	5'-FAM- CGGCAGCACATATACTAAAATTGGAACGA-TAM

Supplementary Table S2: Characteristics of patients with aGVHD

Patient number	Time of aGVHD	Ratings of aGVHD			Grade of aGVHD	Therapy	Outcome
		Skin	Gastrointestinal tract	Liver			
3	14 d	2	2	2	3	steroid	CR
4	12 d	2	1	0	2	steroid	CR
6	55 d	2	0	0	1	steroid	CR
7	12 d	3	0	0	2	steroid	CR
10	20 d	2	0	0	1	steroid	CR
15	12 d	3	0	0	2	steroid	CR
16	15 d	2	0	0	1	steroid	CR
19	16 d	2	0	0	1	steroid	CR
20	18 d	3	2	0	3	steroid	CR
21	28 d	2	0	0	1	steroid	CR
22	20 d	2	0	1	2	steroid+MTX	CR
25	21 d	2	0	0	1	steroid+basiliximab	CR
32	24 d	2	2	2	3	steroid(2 mg/ kg)+basiliximab	NR
34	11 d	3	0	0	2	steroid+basiliximab	CR
39	13 d	2	0	0	1	steroid	CR
44	33 d	0	3	1	3	steroid+basiliximab	CR
47	15 d	2	3	0	3	steroid+basiliximab	CR
49	42 d	2	0	0	1	steroid	CR
60	16 d	2	2	0	3	steroid	CR
61	17 d	2	0	0	1	steroid	CR
63	25 d	2	0	0	1	steroid	CR
75	17 d	2	0	0	1	steroid	CR
102	25 d	2	3	1	3	steroid(2 mg/ kg)+basiliximab	CR
107	12 d	2	1	0	2	steroid	CR
109	20 d	2	0	0	1	steroid	CR
114	14 d	2	0	0	1	steroid	CR
123	20 d	2	0	0	1	steroid	CR
125	17 d	0	1	0	2	steroid	CR
116	8 d	2	0	0	1	steroid	CR
160	30 d	2	0	0	1	steroid	CR
161	15 d	2	0	0	1	steroid	CR
165	21 d	3	1	0	2	steroid+basiliximab	CR
177	25 d	1	4	0	4	steroid+basiliximab	CR
202	18 d	2	0	0	1	steroid	CR
223	9 d	3	0	0	2	steroid+basiliximab	CR

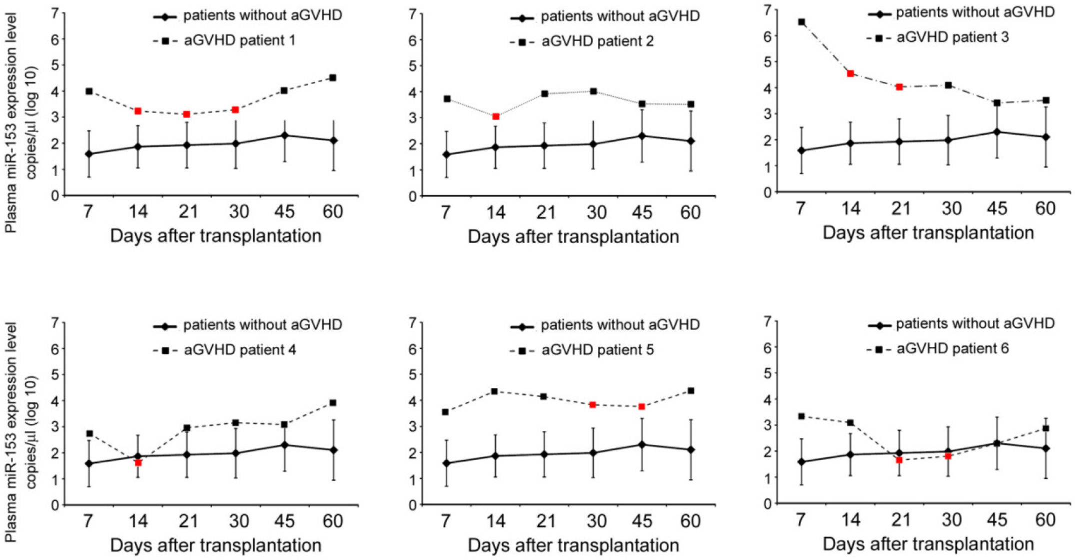

Supplementary Figure S1: Expression profile of plasma miR-153-3p from 6 representative patients with aGVHD after allo-HSCT. miR-153-3p expression in 35 patients without aGVHD are shown as the control. The red points indicate the time of aGVHD occurrence.

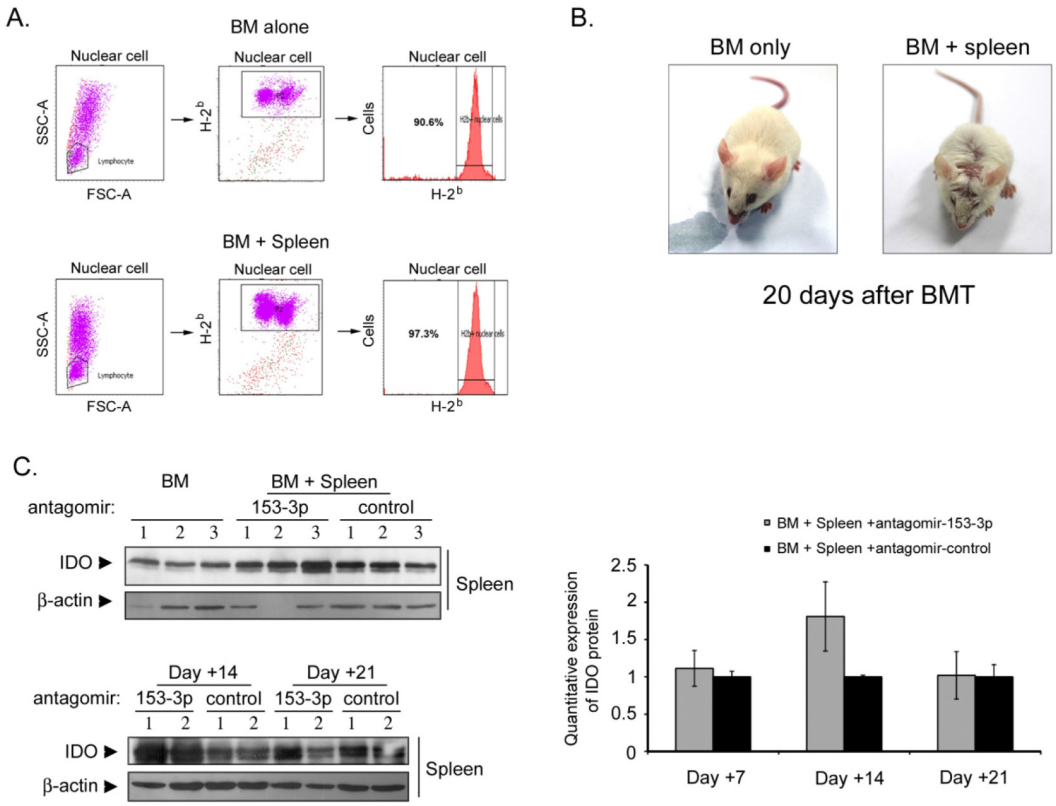

Supplementary Figure S2: A. Flow cytometry analysis of the mouse engraftment at +7 d. Peripheral blood cells were stained with H-2Db (donor mice) antibody. **B.** Clinical signs of the mice that received BM or BM plus spleen cells. **C.** IDO expression levels in the spleen were detected by immunoblotting at +7 d, +14 d, +21 d after allo-HSCT.
